# Current status of African swine fever virus in a population of wild boar in eastern Poland (2014-2015)

**DOI:** 10.1007/s00705-015-2650-5

**Published:** 2015-10-26

**Authors:** Grzegorz Woźniakowski, Edyta Kozak, Andrzej Kowalczyk, Magdalena Łyjak, Małgorzata Pomorska-Mól, Krzysztof Niemczuk, Zygmunt Pejsak

**Affiliations:** Department of Swine Diseases, National Veterinary Research Institute, Partyzantów 57 Avenue, 24-100 Puławy, Poland; National Veterinary Research Institute, Partyzantów 57 Avenue, 24-100 Puławy, Poland

**Keywords:** African swine fever virus, Epidemiology, Diagnosis, Wild boar

## Abstract

African swine fever virus (ASFV) was detected in wild boar in eastern Poland in early 2014. So far, 65 cases of ASFV infection in wild boar have been recognised. The methods used for ASFV detection included highly specific real-time PCR with a universal probe library (UPL), enzyme-linked immunosorbent assay (ELISA), and an immunoperoxidase test (IPT) for identification of anti-ASFV antibodies. The positive ASF cases were located near the border with Belarus in Sokółka and Białystok counties. Some of the countermeasures for disease prevention include early ASF diagnosis by ASFV DNA identification as well as detection of specific antibodies by systematic screening. The aim of this study was to assess the current ASF status in a Polish population of wild boar during the last two years (2014-2015).

African swine fever (ASF) is a contagious viral disease affecting swine, wild boar, warthogs, and other hosts belonging to family *Suidae* [2, 4, 5, 7, 16, 19]. The disease is caused by African swine fever virus (ASFV), the sole member of the family *Asfarviridae*. ASF is a notifiable disease that seriously affects local and international trade of swine and processed meat products [19, 21]. ASF was first diagnosed in Europe (Portugal) at the end of 1950. The disease was then found in Malta, Italy, France, Belgium and the Netherlands [2, 5, 6, 9–13]. Due to successful biosecurity regulations, African swine fever virus (ASFV) was then eradicated from most European regions at the beginning of 1990. However, it still remains endemic in Sardinia and sub-Saharan Africa [10, 11, 22]. African swine fever (ASF) became exceptional in 2007, when the virus reached Poti docks in Georgia with contaminated feed for pigs [13–16, 20]. This event has irreversibly affected the international trade of pig meat. ASF was then identified in Armenia, Azerbaijan, and the Caucasus region of the Russian Federation (RF) [12, 14]. Subsequently, the virus spread to Belarus, Ukraine, Estonia, Latvia and Lithuania [12, 20–22]. In Poland, ASFV was detected for the first time in February 2014 in dead wild boar in Sokółka county [17, 18]. Previously, we described the ASF epidemiological situation in August 2014 and described 14 cases and two outbreaks in pigs. However, the virus continued to spread among wild boar within Sokółka and Białystok counties, with two peaks of infection in July and August and in November and December [18]. Since there is not a commercially available vaccine against ASF, one of the most important measures for disease prevention is control of wild boar reproduction. However, even if a vaccine were available, it might be used only for domestic pigs and not wild boars. Additionally, pigs from small-scale holdings are prone to come in contact with potentially infected wild boars due to low-biosecurity conditions [17, 18]. There are very few diseases for which vaccines are used in wild animals. Also, the diagnosis of ASF is exceptionally important to control the spread of infection among populations of wild boar and pigs. Therefore, the role of the National Reference Laboratory (NRL) for diagnosis of ASF seems to be essential to cope with the current situation caused by this devastating disease of wild boars and pigs. The methods applied by the NRL include real-time PCR with application of a universal probe library (UPL), enzyme-linked immunosorbent assay (ELISA), and an immunoperoxidase test (IPT) for anti-ASFV identification of antibodies. Further genotyping using p72 gene sequencing may also provide additional information about the genotypes of ASFV isolates [3, 9]. It is important to emphasize that viral DNA may only be detected during acute infection of wild boar and swine. The DNA concentration may be high among animals suffering from acute infection or in carcasses of animals that died of acute ASF [8, 16]. Viremic animals suffering from acute ASF are frequently negative for antibodies by either ELISA or IPT for a period of time. These animals require at least 7-10 days from the time of infection to develop an antibody immune response that is detectable by ELISA or IPT [16]. The aim of this study was to summarise the current status of ASFV in a population of wild boar in Poland for the last 17 months since the first diagnosed case.

All stages of processing and sample preparation for diagnosis of ASF were performed in a biosafety level 3 laboratory (BSL-3) by qualified staff and supervisors. The standard Ba71V strain with a titer of 10^8^ 50 % hemadsorption units (HADU_50_) was used as a positive control. It was kindly provided by the European Union Reference Laboratory (URL) for ASF (CISA-INIA, Valdeolmos, Spain). Until June 17, 2015 (starting from January 1, 2014), a total of 29,533 samples of blood, internal organs (spleen, lungs, kidneys, tonsils and bone marrow) from hunted or dead wild boar (*Sus scrofa*) were collected, corresponding to 22,095 individual animals. The samples were sent to the NRL for ASF at the NVRI, Pulawy, Poland, for ASFV monitoring. The sections of tissues were processed as 10 (w/v) homogenates in phosphate-buffered saline (PBS) and then used for DNA extraction. Blood clots from dead wild boar were used to obtain serum samples, which were prepared as 1:2 or 1:80 dilutions and then examined by ELISA and IPT, respectively.

Total DNA was extracted from 200-µL samples from infected cell culture, peripheral blood or tissue homogenates using a High Pure PCR Template Preparation Kit, following the manufacturer’s procedure (Roche Diagnostics, Basel, Switzerland). The final elution was done with 50 µL of sterile nuclease-free water. The extracted DNA was stored at -20 °C until further analysis.

Real-time PCR with an UPL was conducted in eight 0.2-ml optical tubes in an MX3005P real-time PCR system (Stratagene, Agilent Technologies Inc., Santa Clara, CA, USA). The primers ASF-VP72-F and ASF-VP72-R and probe UPL#162 used for this method were complementary to the conserved VP72 sequence region of ASFV. The primer sequences and reaction conditions were consistent with the previously described protocol [8]. Briefly, real-time PCR was conducted using a LightCycler 480 Probes Master kit (Roche Applied Science, Basel, Switzerland) in a final volume of 20 µL. The reaction mixture contained 1x-concentrated LC480 Probes Master PCR Mix, 0.4 µM each ASF-VP72-F and ASF-VP72-R primer and 0.1 µM UPL#162 probe. The thermal programme was as follows: 5 min at 95 °C (initial denaturation), followed by 40 cycles at 95 °C for 10 s (exact denaturation) and 60 °C for 30 s (primer annealing and PCR product elongation). The fluorescence signal was collected during the primer-annealing and elongation step of each cycle using the FAM channel (excitation λ = 495 nm, emission λ = 520 nm). A fluorescent curve with a threshold cycle value (Ct) lower than 38 was considered a positive result.

Enzyme-linked immunosorbent assay (ELISA) was conducted to detect specific anti**-**ASFV antibodies in sera collected from wild boar. The antibodies were detected using an Ingezim PPA Compac 1.1.PPA K3 ELISA Kit (Ingenasa, Madrid, Spain). The assay was assumed to be valid if the optical density (OD) ratio of the negative control (N_c_) to the positive control (P_c_) was equal to or greater than 4. The positive cutoff was calculated as N_c_ - (N_c_ - P_c_) × 0.5, while the negative cut off was calculated as N_c_ - (N_c_ - P_c_) × 0.4. The serum samples were considered positive if the average of their OD values was lower than the positive cutoff. The samples were considered negative if the average OD was higher than the negative cutoff. The sera that were considered doubtful had an average OD between the calculated positive and negative cutoff values. In general, the P_c_ value reached ~0.1 ± 0.02, while Nc reached ~1.4 ± 0.03. The declared sensitivity and specificity of this ELISA were between 95 % and 98 %.

The IPT was conducted using fixed Vero cells infected with Ba71V ASFV in 96-well plates as described elsewhere [10].

The results obtained for positive ASFV cases were compared to the total number of wild boar examined in 2014 and 2015 and analyzed using a two-tailed difference test. The significance level was α = 0.05. Calculations were made in Microsoft Excel ver. 2007 (Microsoft, Redmond, Washington, USA).

All 65 diagnosed ASFV cases were located near the Belarusian border in Podlaskie voivodeship within Białystok and Sokólka counties. The case that was most distant from the border was C58, located close to the town of Krukowszczyzna (Fig. 2A, Table 1). The distances for particular cases are shown in Fig. 2A. On the February 14, 2014, the first case was reported in dead wild boar from the town of Grzybowszczyzna in Sokólka County (Fig. 1, Table 1). The C_T_ value obtained with homogenates of liver and lungs was 30.63 ± 0.09. Due to the sample type, it was not possible to perform serological assays (ELISA and IPT). Next, four cases in wild boar (C2, C3, C4, C5) were identified within the same county. The C_T_ values obtained with these samples collected from affected animals ranged from 22.49 ± 0.23 in a wild boar from the town of Łosiniany (C4) to 27.45 ± 5.11 in samples collected in Rudaki (C3) (Table 1, Figs. 1 and 2). The presence of anti-ASFV antibodies was detected in blood of wild boar from C2 (OD = 0.81 ± 0.00) and C4 (OD = 0.96 ± 0.00). These cases were noted between February and the end of June 2014. At the same time, C6 was diagnosed in wild boar from Bobrowniki in Sokółka County (Table 1). The presence of specific anti-ASFV antibodies in the blood of wild boar from C6 was confirmed by ELISA (OD = 0.52 ± 0.00) and IPT. In the period between July and August, eight new cases (C7-C14) were identified. These were located mainly in Białystok (C7-C12, C14) and Sokółka counties (C11, C13) (Fig.1, Table 1). The observed C_T_ values from C7, C8 and C9 ranged from 28.44 ± 0.00 (C8) to 30.35 ± 0.00 (C9) in the samples from carcasses found in Skroblaki, Łużany and Wiejki/Zubry (Table 1, Fig. 2B). These values were considerably lower than those obtained from C10-C14. The mean C_T_ values are showed in Fig. 2B to present the relative concentration of viral DNA in particular cases. In the time period from September 2014 to December 2014, 16 new cases (C15-C30) were identified within Białystok and Sokółka counties. The observed mean C_T_ values ranged from 20.57 ± 1.32 (C21) to 38.5 ± 0.00 (C20). Further analysis of these case by ELISA showed the presence of specific antibodies in wild boar blood collected from C20 (0.88 ± 0.01), C23 (1.03 ± 0.00) and C26 (0.31 ± 0.00). In other investigated cases, specific antibodies were not detected. The beginning of January 2015 brought new ASF cases (C31) located in Raduin in Białystok County. Between February and June 2015, 33 new ASFV cases (C32-C65) were identified in hunted or dead wild boar from Białystok and Sokólka counties, but case C43 was confirmed for the first time in Hajnówka district, 4 km from the Belarusian border (Table 1, Fig. 2A). The C_T_ values were the highest for C40 (21.45 ± 0.00).

**Table 1:** Positive results for samples collected from wild boar (cases) between 2014 and 2015 using real-time PCR UPL, ELISA and IPT assays

Case (C)/number	Location (nearest town)	Location (county)	Collection date	Sample origin (D-dead, H-hunted wild boar)/tissue	Mean real-time PCR cycle threshold value (C_T_) ± SD	ELISA result/OD ± SD	IPT result
C1	Grzybowszczyzna	Sokółka	18.02.2014	D/bone	30.63 ± 0.09	N/A	N/A
C2	Ozierany Wielkie	Sokółka	25.03.2014	D/blood and lung	25.12 ± 8.22	+/(0.81 ± 0.00)	-
C3	Rudaki	Sokółka	19.05.2014	D/blood and spleen	27.45 ± 5.11	+/-/(1.15 ± 0.00)	N/A
C4	Łosiniany	Sokółka	29.05.2014	D/blood and lung	22.49 ± 0.23	+/(0.96 ± 0.00)	N/A
C5	Słoja	Sokółka	24.06.2014	D/spleen	24.46 ± 3.20	N/A	N/A
C6	Bobrowniki	Białystok	30.06.2014	D/blood and spleen	27.66 ± 4.54	+/(0.52 ± 0.00)	+
C7	Łużany	Białystok	04.07.2014	D/bone	28.44 ± 0.00	N/A	N/A
C8	Wiejki/Zubry	Białystok	08.07.2014	D/bone	28.32 ± 0.00	N/A	N/A
C9	Skroblaki	Białystok	15.07.2014	D/bone	30.35 ± 0.00	N/A	N/A
C10	Gródek-Wiejki	Białystok	29.07.2014	D/bone	25.28 ± 0.00	N/A	N/A
C11	Jałówka-Łupianka Nowa	Białystok	29.07.2014	D/bone	22.72 ± 0.00	N/A	N/A
C12	Wiejki-Zubry	Białystok	30.07.2014	D/blood and spleen	21.42 ± 2.12	-	-
C13	Horczaki Górne	Białystok	21.08.2014	D/blood and spleen	28.93 ± 5.61	-	-
C14	Kolonia Mostowlany	Sokółka	24.08.2014	D/spleen and kidney	25.55 ± 1.18	N/A	N/A
C15	Zaleszany Kolonia	Białystok	12.09.2014	D/spleen and kidney	22.55 ± 0.00	N/A	N/A
C16	Straszewo	Białystok	22.09.2014	H/blood	22.26 ± 0.00	-	-
C17	Nowosady Kolonia	Białystok	04.10.2014	D/blood and kidney	23.56 ± 1.84	-	-
C18	Pieszczaniki	Białystok	07.10.2014	D/blood	27.07 ± 3.51	-	-
C19	Wyżary	Białystok	20.10.2014	D/bone	20.83 ± 0.00	N/A	N/A
C20	Zaleszany	Białystok	11.11.2014	H/blood	38.50 ± 0.00	+/(0.88 ± 0.01)	+
C21	Wiejki	Białystok	21.11.2014	D/blood and spleen	20.57 ± 1.32	-	-
C22	Ostrów Południowy/Górany	Białystok	24.11.2014	H/blood	24.89 ± 0.00	-	-
C23	Mieleszki	Białystok	30.11.2014	H/blood	32.17 ± 0.00	+/(1.03 ± 0.00)	+
C24	Piłatowszczyzna	Sokółka	30.11.2014	H/lung	33.20 ± 0.00	N/A	N/A
C25	Podłaźnisko	Białystok	02.12.2014	D/spleen and kidney	20.73 ± 2.40	N/A	N/A
C26	Mostowlany	Sokółka	05.12.2014	H/blood	40.00 ± 0.00	+/(0.31 ± 0.00)	+
C27	Piłatowszczyzna	Białystok	06.12.2014	H/blood and lung	25.33 ± 0.00	-	-
C28	Nowosady	Sokółka	06.12.2014	H/lung	34.09 ± 0.00	N/A	N/A
C29	Grzybowce/Skroblaki	Białystok	06.12.2014	H/lung	25.06 ± 0.38	N/A	N/A
C30	Podłaźnisko	Białystok	23.12.2014	D/blood, liver, spleen and kidney	21.86 ± 0.00	-	
C31	Radunin	Białystok	16.01.2015	D/spleen	17.64 ± 0.00	N/A	N/A
C32	Zadworzany	Sokółka	02.02.2015	D/blood, spleen kidney	26.53 ± 7.50	-	-
C33	Piłatowszczyzna	Sokółka	29.01.2015	H/blood, and spleen	24.00 ± 0.00	-	-
C34	Cisówka	Sokółka	03.02.2015	D/kidney	24.45 ± 0.00	N/A	N/A
C35	Budy	Białystok	05.02.2015	D/spleen	26.21 ± 5.20	N/A	N/A
C36	Wierzchlesie	Białystok	13.02.2015	D/bone	29.86 ± 0.00	N/A	N/A
C37	Bielewicze	Białystok	15.02.2015	D/bone	36.28 ± 0.00	N/A	N/A
C38	Kondratki	Sokółka	16.02.2015	D/bone	31.46 ± 0.00	N/A	N/A
C39	Kolonia Bachury	Białystok	02.03.2015	D/bone	27.86 ± 0.00	N/A	N/A
C40	Kondratki	Białystok	07.03.2015	D/spleen	21.45 ± 0.00	N/A	N/A
C41	Majdan	Białystok	10.03.2015	D/spleen and kidney	24.59 ± 0.00	N/A	N/A
C42	Bielewicze	Białystok	15.03.2015	D/blood and spleen	24.52 ± 1.77	+/(0.85 ± 0.01)	+
C43	Narewka	Hajnowka	19.03.2015	D/blood and spleen	27.93 ± 6.31	-	-
C44	Kolonia Cisówka	Białystok	25.03.2015	D/bone	30.80 ± 2.70	N/A	N/A
C45	Biały Ług	Skololka	25.03.2015	D/blood, spleen, tonsils	24.48 ± 2.49	+/-/(1.15 ± 0.00)	-
C46	Kolonia Cisowka	Białystok	30.03.2015	D/spleen	21.37 ± 0.00	N/A	N/A
C47	Bielewicze	Białystok	30.03.2015	D/lung	30.62 ± 2.58	N/A	N/A
C48	Straszewo	Białystok	1.04.2015	D/bone	26.20 ± 0.00	N/A	N/A
C49	Cisowka	Białystok	02.04.2015	D/bone	29.71 ± 0.00	N/A	N/A
C50	Kruglany	Sokółka	05.04.2015	D/blood, spleen and kidney	26.32 ± 0.00	-	N/A
C51	Straszewo	Białystok	09.04.2015	D/bone	37.74 ± 0.00	N/A	N/A
C52	Wierzchlesie	Sokółka	10.04.2015	D/spleen and tonsils	23.36 ± 0.00	N/A	N/A
C53	Puciłki	Sokółka	11.04.2015	D/blood, spleen and tonsils	25.30 ± 0.00	-	N/A
C54	Borsukowizna	Sokółka	14.04.2015	D/spleen and tonsils	38.60 ± 0.00	N/A	N/A
C55	Straszewo	Białystok	15.04.2015	D/tonsils	26.52 ± 0.80	N/A	N/A
C56	Juszkowy Gród	Białystok	16.04.2015	H/blood	34.90 ± 0.00	+/(0.26 ± 0.00)	+
C57	Kolonia Mieleszki	Białystok	18.04.2015	H/blood	32.14 ± 0.19	+/(0.11 ± 0.00)	+
C58	Krukowszczyzna	Białystok	27.04.2015	D/spleen and kidney	26.94 ± 0.00	N/A	N/A
C59	Łaźnisko	Sokółka	08.05.2015	D/spleen and kidney	24.02 ± 0.82	N/A	N/A
C60	Dzierniakowo	Białystok	13.05.2015	D/lung	23.44 ± 0.83	N/A	N/A
C61	Nowosady	Białystok	20.05.2015	D/spleen	23.02 ± 0.04	N/A	N/A
C62	Nowe Trzciano	Sokółka	21.05.2015	D/blood and spleen	27.23 ± 0.00	-	N/A
C63	Cisówka	Białystok	22.05.2015	D/bone	34.61 ± 0.00	N/A	N/A
C64	Planty	Białystok	1.06.2015	D/bone	29.30 ± 3.05	N/A	N/A
C65	Dzierniakowo	Białystok	2.06.2015	D/bone	25.12 ± 0.00	N/A	N/A

The case number, origin of each sample, localization, collection date and mean cycle threshold (C_T_) values with standard deviation (SD) are given. The ELISA results expressed as optical density (OD) are given. The ELISA and IPT results are provided where applicable. N/A, not applicable; SD, standard deviation. The sera originating from dead wild boar were retrieved from blood clots

**Fig. 1:**
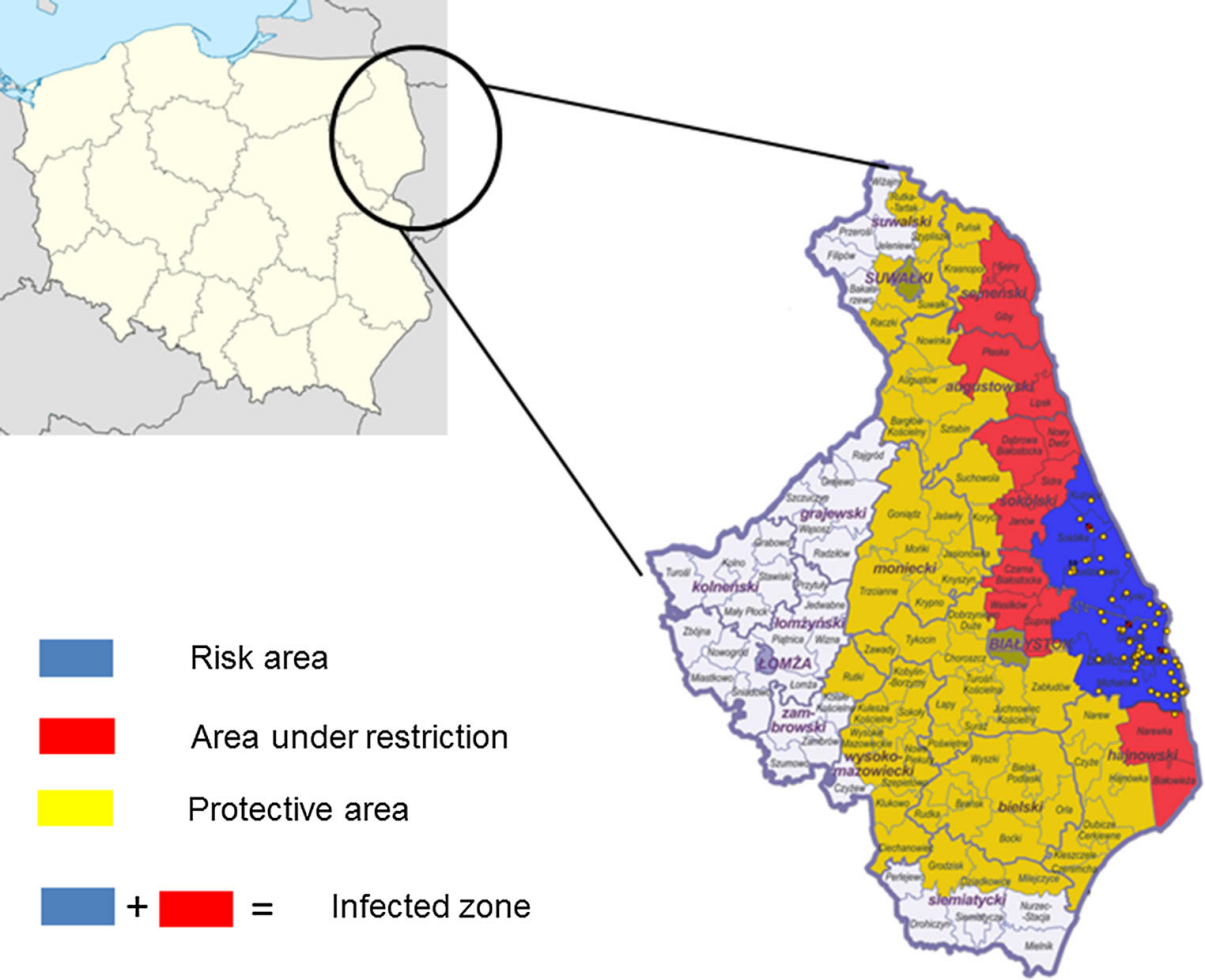
Localisation of ASFV- positive animals. Blue, ASF risk area; yellow, protected area; red, restricted area; blue + red, infected area. The map originates from the resources of the General Veterinary Inspectorate, Warsaw, Poland. (color figure online)

**Fig. 2:**
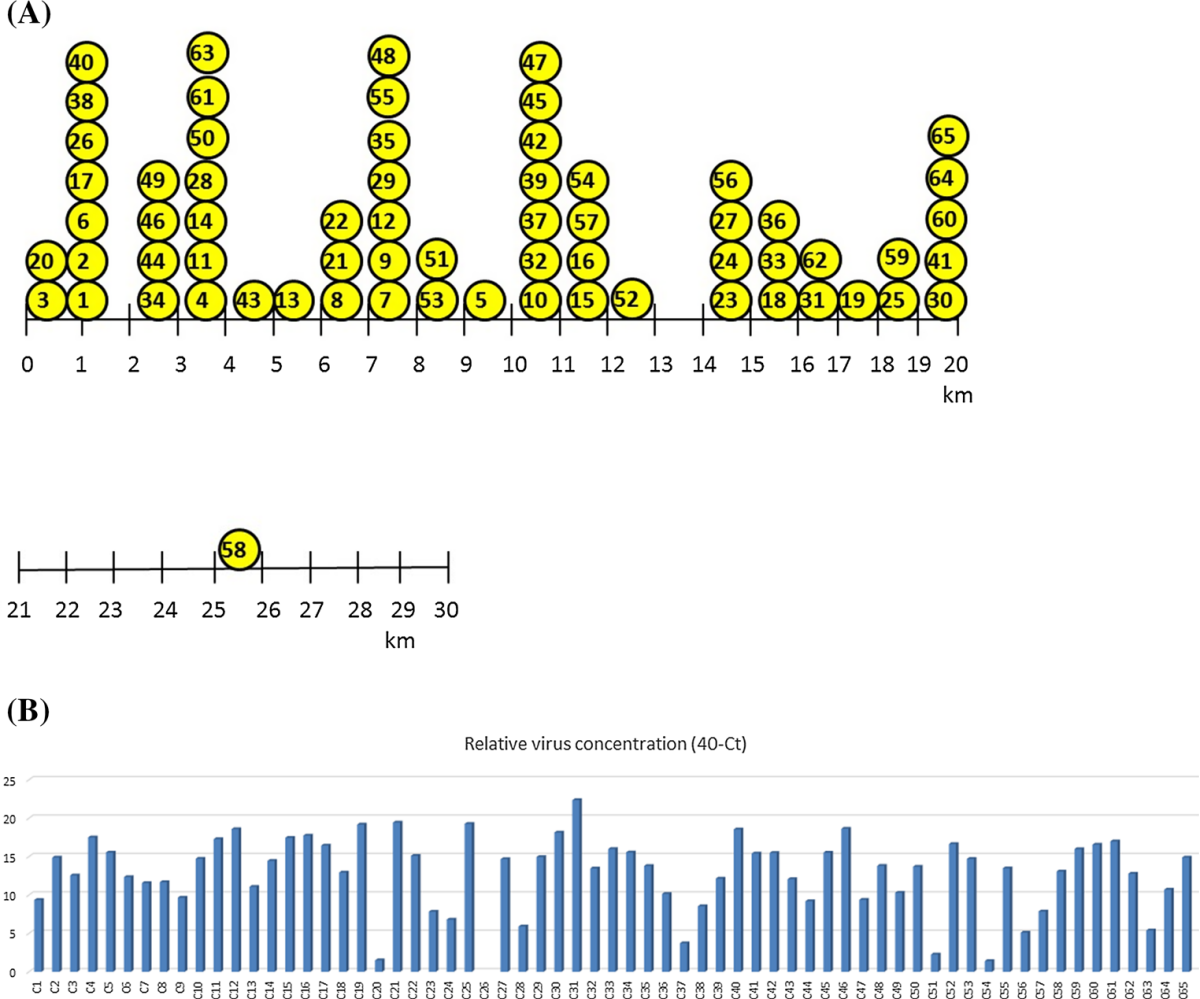
Distance of ASFV-positive animals from the Belarusian border (A) and comparison of mean cycle threshold values (C_T_) from particular cases (B). The graph shows the relative DNA concentration is samples collected from particular cases and outbreaks of ASFV. The C_T_ value for C26 reached 40.00 and is absent from the graph. The localization of particular cases shows a negative correlation between distance and transfer of ASFV infection

The ELISA results showed the presence of anti-ASFV antibodies in wild boar from the cases C2-C4, C6, C23, C26, C42 (0.85 ± 0.01), C45 (1.15 ± 0.00), C56 (0.26 ± 0.00) and C57. However, these results were only confirmed by IPT in C6, C20, C23, C26, C42, and C56-C57 (0.11 ± 0.00; 70 % of ELISA results). Low or negative real-time PCR results were obtained in C20 and C26, but in contrast, these samples were serologically positive. In the case of C42, the high DNA concentration, with a C_T_ value of 24.52 ± 1.77, was consistent with the positive results in ELISA (0.85 ± 0.01) and IPT (Table 1). We found that the vast majority of cases were identified in the vicinity (within 1-10 km) of Belarusian territory (Fig. 2A), with few new ASFV cases occurring more than 11-15 km away from the Belarusian border.

The circulation of ASFV in Poland has national and international consequences for the swine trade and production, especially in the northeastern part of the country [17, 18]. The aim of this study was to investigate the current epidemiological status of ASFV in eastern Poland. The methods used within this study included real-time PCR with a UPL as described previously by Fernaéndez-Pinero et al. [8]. The ASF real-time PCR with the UPL has been shown to be a sensitive and specific technique for the detection of ASFV. Other diagnosis techniques for ASFV include a number of real-time PCR tests [1, 18] and serological assays such as ELISA and IPT [16]. However, it should be noted that specific anti-ASFV antibodies are produced during the late stage of infection. In this study, we report 65 diagnosed cases of ASFV in Poland that occurred near the Belarusian national border. The analysis showed that the affected wild boar were able to migrate from 0.5 to 25 km, but the most frequent distance was 1-10 km. Fortunately, the virus was not detected outside the restricted area, indicating that control measures to limit wild boar reproduction have been successful. A comparison of C_T_ values from real-time PCR assays showed a broad range of values in the context of particular cases. The presented data may be treated as a review of the virus replication cycle or age of carcasses and cannot reveal the real transmission of ASFV itself. It might be especially important in case of samples originating from carcasses of wild boar or bones. However, the comparison of different C_T_ values obtained from the well-standardized method from different cases may at least provide reliable data on the relative concentration of viral DNA. However, due to the different kinds of samples used (bone marrow, internal organs or blood), it was difficult to provide reliable quantification of the virus in these materials. The conducted serological assays including ELISA or IPT facilitated confirmation of real-time PCR results in six ASF cases. However, material for this study was also collected from wild boar carcasses, allowing only viral DNA extraction. The number of analysed samples and ASF cases showed a peak of virus transmission between July and August but also during February and March. Between January and May 2014, analysis of material originating from 6759 wild boar revealed three positive ASFV cases. In the same season of 2015, analysis of 4388 wild boar showed the occurrence of 29 ASFV cases. Statistical analysis at the 95 % confidence level (0.04 % in 2014 and 0.66 % in 2015) showed that the observed change in case number between 2014 and 2015 was significant (*p* < 0.05). These findings need to be extended for the upcoming months or years.

This study shows that ASFV transmission is not as rapid as was previously predicted [4, 16]. However, it still presents a serious economic threat. The virus seems to be continuously transmitted in the wild boar population, but in general, the distances do not exceed 10 km. The conducted diagnostic investigations showed that the majority of the infected wild boars died before the onset of immunity but had a high viral DNA load. This might indicate a rapid progress of wild boar ASFV infection that leads to sudden death. Two exceptions, in cases C20 and C26, suggest that hunted wild boars had survived the initial infection and, in spite the low or negative C_T_ values, remained serologically positive. This could indicate the presence of naturally attenuated ASFV isolates among wild boar population. Therefore, an interesting issue for our future study will be virus isolation from wild boar showing detectable antibody titers. These isolates will be also used to inoculate experimental animals and examine their virulence.
